# Service integration: The perspective of Australian alcohol and other drug (AOD) nurses

**DOI:** 10.1111/inm.12998

**Published:** 2022-03-25

**Authors:** Adam Searby, Dianna Burr, Russell James, Phil Maude

**Affiliations:** ^1^ Institute for Health Transformation School of Nursing & Midwifery Deakin University Geelong Victoria Australia; ^2^ School of Nursing College of Health and Medicine University of Tasmania Hobart Tasmania Australia; ^3^ La Trobe Rural Health School Violet Vines Marshman Centre for Rural Health Research Latrobe University Melbourne Victoria Australia

**Keywords:** delivery of health care, integrated, dual diagnosis (psychiatry), mental health nurses, mental health services, substance abuse treatment services

## Abstract

The recently released Victorian Mental Health Royal Commission report has recommended a shift to integrated treatment, defined as treatment for alcohol and substance use disorders and mental ill health occurring in parallel, rather than distinct systems catering to each need. However, little work has sought to determine the perceptions of nurses working in alcohol and other drug (AOD) treatment towards integrating with mental health services. In this study, we explore the perspectives of specialist AOD nurses towards the integration of mental health and AOD treatment services. Secondary analysis of semi‐structured interviews with Australian specialist AOD nurses (*n* = 46) conducted as part of a wider workforce study in 2019. Data were analysed using thematic analysis and reported using the COREQ guidelines. Of the interviews analysed, six were AOD nurses working in an Australian state that had recently undergone service integration; however, many participants expressed perceptions of service integration. Two key themes are reported in this paper: (i) perceptions of service integration, where AOD nurses participating in our study were concerned that integration would result in the model of care they worked under being replaced by a mental health‐based model that was felt to be highly risk averse, and (ii) experiences of service integration. Concerns about the focus of care as well as the complexity of care differing between the two services demonstrated a contrast in both philosophical approaches to work with consumers and legislative difference in voluntary versus compulsory care provision.

## Introduction

Service integration, here defined as combining alcohol and other drug (AOD) treatment into mental health treatment, is a recommendation of the recent Royal Commission into mental health in Victoria, Australia (State of Victoria 2021). In Australia, treatment for AOD use disorders and mental ill health has traditionally been delivered separately; this has resulted in criticisms that mental health services often overlook AOD use disorders, and AOD services do not have the capacity to manage consumers with severe, low prevalence mental illness presentations (Groenkjaer *et al*. 2017; Munro & Edward, 2008; Searby *et al*. 2017). Although integrated treatment shows promise in managing complex dual diagnosis presentations, research indicates that consumers who seek AOD treatment are often reluctant in entering treatment operated by mental health services for fear of receiving a mental illness diagnosis, becoming enmeshed in the mental health system, or stigma associated with AOD use (Mojtabai *et al*. 2014).

This paper explores the perceptions of nurses working in AOD settings towards integrating with mental health services. The findings from this study were drawn from a larger mixed methods project mapping the AOD nursing workforce in Australia (Searby & Burr 2020). When conducting the qualitative component of this study, several participants described their experiences with, or beliefs around service integration. We considered this data to be worthy of secondary analysis and felt that considering the current recommendations of the Victorian Mental Health Royal Commission (State of Victoria, 2021) and ongoing interest in service integration this paper is timely.

## Background

The Victorian Mental Health Royal Commission commenced in February 2019 with the aim of reporting the status and recommending improvements for a mental health system that was described as ‘broken’ (State of Victoria 2021). The final report found substantial issues in the Victorian mental health system, including poor system design, difficult access, complexity and fragmented service delivery; subsequently 65 reforms to improve Victorian mental health services were recommended. Recommendation 35, *Improving health outcomes for people living with mental illness and substance use or addiction*, was one of two recommendations specifically concerning mental ill health and addiction, and recommends that all mental health services ‘… provide integrated treatment, care and support to people living with mental illness and substance use or addiction’, (State of Victoria 2021, p. 72).

In the mental health context, integrated treatment is defined as the delivery of services for both mental ill health and AOD use in an interwoven fashion; however, there are many variations on how integrated services are provided (Minkoff 2007). Generally, in Australia, mental health services and AOD treatment services have operated separately; this has resulted in a model of ‘serial’ service delivery, where healthcare consumers seeking treatment for mental health are directed to AOD treatment first, or where each service refuses consumers due to acuity in an area (mental illness or alcohol and drug use) that is either perceived as the primary concern or not the specialty of the service (Staiger *et al*. 2010).

Service integration has been a contentious issue in Australia for some time. In 1993, the Victorian Community Managed Mental Health Services (VICSERV) released a report describing a research project conducted in 1988 (McDermott & Pyett 1993), finding several services at the time that were unable to provide comprehensive care to individuals with co‐occurring mental ill health and substance use disorders (dual diagnosis). The report called for a ‘no wrong door’ policy where services accepted all consumers with complex presentations. The notion of accepting all individuals seeking service rather than turning them away (no wrong door) was echoed in a document released by the Victorian Government in 2007, which called for dual diagnosis to become ‘core business’, and for clinicians working in mental health services to be upskilled in managing alcohol and substance use disorders (Victorian Government Department of Human Services 2007). Although not directly calling for integrated services, both seminal documents report the difficulties in managing mental ill health or AOD use in services not specifically targeting these issues.

Existing studies examining the effectiveness of integrated services have either methodological issues or show little improvement of existing separate systems of care (Hobden *et al*. 2018). Despite the lack of quality evidence supporting the integration of mental health and AOD services, the Victorian Government has committed to the delivery of a framework for service integration by mid‐2022, with work commenced on this framework in 2021; services are also expected to develop plans for integration by mid‐2022 (Victorian State Government Department of Health 2021).

Prior attempts at integration have been attempted due to perceived service gaps, and where integration has been attempted, have largely involved ‘horizontal integration’ between services (Edward *et al*. 2012; Flatau *et al*. 2013). The state of Queensland recently moved to an integrated service model, with research conducted on consumer experiences (*N* = 39) finding that ‘warm’ referrals, where contact is made by the referring agency prior to the client entering the service, were appreciated by consumers; however, participants also perceived that physical health and comorbidities were often attributed to mental ill health in an integrated model rather than taking a holistic view of individual circumstances (The University of Queensland Institute for Social Science Research 2018).

A recently released report (Lee & Allsop 2020) interviewed key informants (*N* = 18) from the Australian AOD service sector, finding that co‐occurring mental ill health seen in consumers of AOD treatment services revealed often higher prevalence mental health disorders, in contrast to low‐prevalence mental health disorders seen in mental health services, leading the authors to describe a ‘cultural mismatch’ between services. The report also described a notion of care provision as being holistic rather than integrated, providing care for all mental and physical health needs. This recommendation echoes Weiss *et al*.'s (1992) notion of dual diagnosis being a simplistic term for a very complex healthcare issue. Further, this report expressed concerns that integrated services may result in a dilution of specialist skills utilized in AOD treatment and reduced funding to the AOD treatment stream of services.

Global research has shown that integration of services results in a piecemeal adaptation of service components often considered suboptimal to meet the needs of consumers with co‐occurring disorders (Mauro *et al*. 2016). Adoption of integration is mixed despite a consensus that integration is sorely needed and felt to be aligned to the recovery movement (Davidson & White 2007; Peterson 2013). Guerrero *et al*. (2014) analysed 104 addiction treatment programmes in Los Angeles County, California, found that where increased funding was provided to services, and where organizational climate was more supportive of change integration was often successful; these findings indicate that service integration requires not only funding, but also significant investment in the change process to succeed. Where examples are found of successful service integration in existing literature, a specific cohort is often targeted (for example consumers with human immunodeficiency virus or homeless women with mental ill health), with little data available on the integration of large publicly funded mental health services with AOD treatment providers (Nguemo Djiometio *et al*. 2020; Veysey *et al*. 2005).

In this paper, we identify the definition of integration as ‘treatment of multiple problems with a single practitioner or service’, with the combining of AOD and mental health services a common historical approach to service integration in Australia (Lee & Allsop, 2020, p. 8).

## Aim

The aim of this study was to explore the perceptions of nurses working in drug and alcohol settings towards the integration of mental health and AOD services.

## Methods

### Design

The methodology used for this study was qualitative description. Qualitative description is a methodology that allows the examination of events in the everyday language of the events under investigation, meaning the research produces a report in a similar language to that of the participants (Sandelowski 2000). Qualitative description is a naturalistic design, said to be ‘low inference’, with concepts and themes presented in the language participants use to describe events (Kim *et al*. 2017; Neergaard *et al*. 2009). We used qualitative description of this study to provide an account of participant perceptions and experiences of service integration in their own words, to reflect these accounts truly and accurately.

### Data collection

Participants were recruited through an online survey designed to capture demographic information about AOD nurses across Australia. During this process, survey participants were asked if they wished to participate in a telephone interview, providing contact details for further follow‐up by the research assistant. Complete details of the survey study are described elsewhere (Searby *et al*. 2021). Semi‐structured interviews were conducted by telephone between August and October 2019 by the second author (D.B.), a research assistant with extensive experience collecting data for similar studies. The semi‐structured interview guide was developed after a scan of existing grey literature and checked for content validity by the management committee of the Drug and Alcohol Nurses of Australia (DANA), the peak professional association for nurses with a professional interest in AOD issues.

Participants were asked to agree to a verbal consent script read verbatim by the research assistant, and after consenting, all participants completed their interview. Interviews continued beyond theoretical data saturation to attempt to gather responses from a wide geographical area. Interviews were transcribed verbatim by a professional transcription company and transcripts were analysed using the NVivo software program (QSR International, Version 20).

### Ethical considerations

The original study was reviewed by the relevant university ethical review board prior to data collection (RMIT University College Human Ethics Advisory Network, reference 13‐19/22002). Participants were able to view the Participant Information Form, with a verbal consent script being read by the research assistant prior to the interview commencing. All participants who expressed an interest in participating in an interview verbally consented to taking part in the study, with no participants requesting the interview be stopped or their data withdrawn. Any identifying information, such as health service names or local areas, was removed after transcription and are reported generically in this paper. All data were stored on a password protected cloud server (Syncplicity).

### Data analysis

Data were analysed using Braun and Clarke’s (2006) method of thematic analysis. This process outlines six steps in analysing qualitative data: familiarization with the data, generating initial codes, searching for themes, reviewing themes, defining and naming themes, and producing the report. The first two phases of data analysis were conducted by all authors independently, with the authors then meeting to review and define themes collaboratively; during this process, each author was required to discuss their rationale for each code, and final codes were developed when agreement was reached on themes (Hemmler *et al*. 2020).

### Validity and reliability

This study is reported in line with the Consolidated Criteria for Reporting Qualitative Research (COREQ) checklist (Tong *et al*. 2007). During the semi‐structured interview process, extensive notes were made by the research assistant concerning perceptions of the interview, which were later discussed at length with the research team, and documentation for all decisions made during the coding process was retained to ensure transparency, dependability and conformability (Mealer & Jones, 2014). Transcripts were independently checked with the audio recording by research team members other than the interviewer to ensure accuracy, further ensuring the dependability of the study. Transferability was attained by using a purposive sample of AOD nurses who had experience in healthcare services providing treatment for alcohol and substance use disorders.

## Findings

Forty‐six (*n* = 46) transcripts were analysed for this study. Participants were mostly female (*n* = 35), with 11 males participating, and with representation from all states and territories in Australia. Participant demographics, including location, are shown in Table 1. Two key themes emerged from the data analysis process: (i) perceptions of service integration, and (ii) diverse services. The key themes and sub‐themes of the data are shown in Table 2.

**TABLE 1:** Participant demographics

Participant	Gender	Region	Position	Integrated service
Participant 1	Female	Northern Territory	Clinical Nurse	
Participant 2	Female	New South Wales	Government	
Participant 3	Female	South Australia	Education	
Participant 4	Female	Australian Capital Territory	Clinical Nurse	
Participant 5	Female	Victoria	Nurse Practitioner	
Participant 6	Male	New South Wales	Clinical Nurse	
Participant 7	Female	New South Wales	Nurse Manager	
Participant 8	Male	New South Wales	Nurse Practitioner	
Participant 9	Female	New South Wales	Nurse Practitioner	
Participant 10	Female	New South Wales	Clinical Nurse	
Participant 11	Female	New South Wales	Clinical Nurse	
Participant 12	Female	South Australia	Clinical Nurse	
Participant 13	Female	Western Australia	Clinical Nurse	
Participant 14	Male	Western Australia	Clinical Nurse	
Participant 15	Female	New South Wales	Clinical Nurse	
Participant 16	Female	Queensland	Nurse Practitioner	✓
Participant 17	Female	Queensland	Nurse Practitioner	✓
Participant 18	Female	New South Wales	Clinical Nurse	
Participant 19	Female	Tasmania	Education	
Participant 20	Male	Northern Territory	Clinical Nurse	
Participant 21	Male	South Australia	Nurse Practitioner	
Participant 22	Male	Queensland	Clinical Nurse	
Participant 23	Female	New South Wales	Clinical Nurse	
Participant 24	Male	Victoria	Clinical Nurse	
Participant 25	Female	New South Wales	Clinical Nurse	
Participant 26	Female	New South Wales	Clinical Nurse	
Participant 27	Female	New South Wales	Clinical Nurse	
Participant 28	Female	New South Wales	Clinical Nurse	
Participant 29	Female	Queensland	Nurse Manager	✓
Participant 30	Female	Western Australia	Clinical Nurse	
Participant 31	Female	New South Wales	Clinical Nurse	
Participant 32	Female	New South Wales	Clinical Nurse	
Participant 33	Male	Queensland	Nurse Practitioner	✓
Participant 34	Female	New South Wales	Nurse Manager	
Participant 35	Female	Victoria	Nurse Practitioner	
Participant 36	Female	New South Wales	Clinical Nurse	
Participant 37	Female	New South Wales	Nurse Manager	
Participant 38	Female	Australian Capital Territory	Nurse Practitioner	
Participant 39	Female	Queensland	Clinical Nurse	✓
Participant 40	Male	New South Wales	Nurse Manager	
Participant 41	Male	Queensland	Nurse Manager	✓
Participant 42	Female	New South Wales	Nurse Practitioner	
Participant 43	Male	New South Wales	Clinical Nurse	
Participant 44	Female	New South Wales	Education	
Participant 45	Female	New South Wales	Clinical Nurse	
Participant 46	Female	New South Wales	Clinical Nurse	

**TABLE 2:** Themes and sub‐themes

Themes	Subthemes
Perceptions of service integration: this theme explores the perceptions of participants toward integrating AOD and mental health services	Negative perceptions of service integration – stigma toward consumers using AOD services Negative perceptions of service integration – service integration happens at the expense of AOD services Negative perceptions of service integration – risk aversity The need for service integration – improving care to consumers
Experiences of service integration: this theme presents the narratives of those who have experienced service integration	

### Perceptions of service integration

In the first key theme, participants expressed their perceptions of integrating mental health and AOD services by affirming the idea or opposing it; for some negative perceptions were expressed regarding service integration; this sentiment was related to the stigma expressed towards consumers who use AOD services, and the perception that service integration occurred at the expense of AOD services. For others it was felt that service integration was needed to improve care to consumers.

#### Negative perceptions – stigma towards consumers using AOD services

Several participants spoke of negative perceptions of service integration. Primarily, these perceptions related to stigma; participants felt that consumers who used AOD services were often more stigmatized than those who used mental health services:

We have a lot of dual diagnosis clients in [AOD] and Mental Health do have clients that use substances to quieten their mind [to] self‐medicate… I have a client who comes and sees me who has cirrhosis. I requested him to go and see a doctor at the local ED Department. ED don’t see him because they view him as just that junkie, just that drunk. This gentleman then has an acute exacerbation of his physical health, but it’s not seen as important because AOD services come under mental health. (Participant 17)

Participants felt that this stigma was detrimental to service provision and acceptance of AOD as a legitimate healthcare setting. The following participant describes the sentiment that mental health consumers often experience empathy, whereas those seeking support for AOD use are often stigmatized due to the notion of their needs for care being due to a conscious choice:

When you’re connected with mental health, [consumers can be seen as], “the poor things. This has happened to them.” In drug and alcohol, it’s quite often considerable stigma and discrimination about “well, you’ve brought that on yourself,” (Participant 6)

One participant extended this concept, reporting a perception that mental health often received a greater share of funding due to public awareness and the politicization of mental health funding:

My concern has always been whilst being connected with mental health is that we are not the same hot political potato that mental health is, therefore our budgets are much more limited, and we end up with much fewer resources than what mental health do. I prefer us to be separate entities focusing on our specialty. (Participant 6)

Another participant went further, describing AOD as the ‘younger brother’ who only received attention and funding when service integration was discussed as a concept. Several participants also reported variations on this theme, where the perception was that AOD was a poorly funded cousin of mental health services:

It’s good to have that focus but what I see is over time is that we’re the younger brother that only gets brought out at Christmas time when you’re integrated into mental health. (Participant 33)

#### Negative perceptions – service integration happens at the expense of AOD services

Participants expressed the opinion that service integration was of great benefit to mental health services but at the expense of AOD services. This perception involved the perceived funding imbalance and the notion that AOD was several ‘rungs’ under mental health in a theoretical treatment hierarchy. Overall, participants described a fear that this situation would continue under service integration models:

[For] 5 years, there has been quite a heavy push to have AOD services structured underneath Mental Health Services, and I say underneath because even though words such as integration are used, really what I see is a lot of those AOD services have been positioned low in the pecking order as far as priority or interest or development or funding for the future. It certainly has a benefit to one party and not much to the other. (Participant 41)

Participants described the differences in consumer presentations, expressing that mental health services often see severe relapse of low prevalence mental illness with consumers treated under compulsory orders, whereas AOD services predominantly see voluntary consumers with trauma, personality disorder and acquired brain injuries. Like consumers presenting to mental health services, there is often a high degree of physical comorbidity; however, this was felt to be different to what is seen in mental health services:

We need to have a group of nurses who are trained to look after complex trauma, personality disorder, brain injury, post‐traumatic stress disorder and you know let's not forget the fact that drug and alcohol clients are very unwell, and we should be looking after people's livers and their blood borne viruses and their burnt‐out oesophagus and everything else. That's not coming from mental health and where is the drug and alcohol basic training? (Participant 40)

As the above account indicates, participants felt that specific skills were required to provide comprehensive care to consumers of AOD services. Many felt that the nature of contemporary mental health services, providing compulsory care to those with low prevalence mental illness, did not provide the requisite skills for working in AOD settings.

#### The need for service integration – improving consumer care

In contrast, some participants spoke of integration as being necessary, often in relation to perceived shortfalls in staff capability to handle presentations of mental ill health in AOD services. These comments largely related to the need to improve capacity to care for consumers who had deteriorating mental health, and a perceived need for AOD nurses to hold these skills. Participants also reflected that caring for consumers with AOD use disorders was often predominant in mental health services:

I think that [integration] does help because… there is so much dual diagnosis and crossover for AOD clients that I have come across in the [region] anyway. (Participant 1)

This comment was reflected by another participant, who felt that the prevalence of dual diagnosis meant a background in mental health was a prerequisite for being an effective AOD nurse:

Because the comorbidity of mental health with drug and alcohol is so high that they go hand in hand, I don’t think you can really treat the alcohol and drug stuff without knowledge of how to support [consumers with] their mental health. (Participant 38)

One participant noted that AOD nurses came to the specialty from a wide variety of settings, bringing skills they felt were valuable in managing physical comorbidities. However, the participant also felt that specific skills were needed to manage mental ill health:

… in our withdrawal service the nurses there, one of them is an intensive care nurse, so she's all over the withdrawal stuff… we've got a couple of nurses with midwifery … it's great drawing those nurses from all those areas. I just do think we need to develop more around the boundary stuff, recognising some of the mental health stuff. (Participant 16)

Another participant echoed that the need for experience in mental health was important to work in AOD settings, particularly when working with consumers who were experiencing a deterioration in their mental health:

I think having experience in other areas is important, particularly maybe mental health skills, you know, being able to recognise deterioration either in somebody’s mental health or their physical health is important. (Participant 37)

As mentioned in the background of this paper, there is often a perception that the service gap between mental health and AOD services means that services can fail to provide comprehensive care for consumers. A participant reflected the current ‘state of play’ with non‐integrated services:

To me there is this very pronounced grey area between drug and alcohol and mental health, and often there’s lack of integrated care or even just appropriate communication between both settings… sometimes they’re separate management hierarchies, sometimes they have good relationships between the services, but sometimes they have bad relationships between each service. (Participant 43)

Finally, as one participant noted, the concept of integrating services should occur where there is benefit for the consumer, although recognizing that both services are distinct:

I recognise that mental health and drug and alcohol are separate yet there is a point of overlap, and we should overlap where we can for the sake of the patient… I think what that does is highlights to mental health that AOD is different but also to AOD that you need to stop being so fearful that you are going to get absorbed by mental health. (Participant 7)

#### Negative perceptions – risk aversity

In addition to the differences in consumer presentations outlined above, participants described significant diversity between AOD and mental health services. Often related to the core functions of each service, participants felt that these differences would be detrimental to the provision of AOD care. Primarily, this perception was related to risk; several participants expressed an opinion where AOD services had a greater tolerance of risk than mental health services. As the following participant described, this approach was often considered consumer centred in AOD settings:

… I think sometimes mental health gets what I call risk averse. I think some people you have to let toddle, and sometimes they will fall over a bit, and you can't protect them, you know. You can give them the information, and two steps forward, a step back, but they'll get to the end of it. And I think the model is about empowering and recovering … (Participant 16)

Another participant reinforced the notion that mental health settings were risk averse, describing ‘conditions’ on admission and treatment that were considered detrimental to the clinical relationship in AOD settings:

On the mental health side of things, that’s a culture that is very risk adverse, very legislated or controlled… there’s a lot of conditions around relationships with consumers in that area. I suppose what I see in the AOD area is that area is probably more open area in terms of its relationships with consumers and probably more patient or client focused, and probably a fairer clinical relationship I see between clinicians and people. Because when you’re using principles like harm minimisation and balancing that with what the person wants to do or why they have come to your service, it’s a lot more person centred. I like that service a hell of a lot more. (Participant 41)

Many participants felt that the perception of addiction as a ‘moral failing’ affected the funding arrangements of AOD services, often due to them being considered less important than acute mental health services. Further, it was felt by some participants that consumers specifically seeking AOD services did not want to use mental health or integrated services:

The community at least has some quite definite ideas around whether they want to walk in a door that’s for an AOD service or a mental health service. One of the stigmas that we see is that a lot of people come to an AOD service say, “this is what I think my problem is and this is why I want help, I’ve already decided that myself.” They don’t want to be labelled or aligned with the idea that they have a mental illness as such or seeing that service… But many people come to our service because they identify AOD. They identify [with] the AOD service as that makes sense to them. (Participant 41)

### Experiences of service integration

There were several participants who had experienced previous attempts at service integration or were currently involved in the active integration of AOD and mental health services. Overall, participants described substantial challenges where services had been integrated, often perceived to be due to both a failure to recognize their specialist skills in working with consumers who use AOD, and the importance of AOD treatment in mental health services:

I work within a mental health service, so my speciality is drug and alcohol within mental health… I've been in this role for nearly 10 years; it's only just been made a substantial position, and in doing that, they've reduced the [hours] and made it more than double the client load. It's all about a capacity‐building model, so what will happen is you'll get someone who has a drug and alcohol portfolio with very little drug and alcohol skills. (Participant 13)

For some participants, service integration meant that their previous positions had become untenable. Often, this was due to integrated services wanting nursing staff to have an educational or professional background in mental health. For those who had worked as AOD nurses for some time, this was a cause of anxiety or loss of previously stable work, and in the following case, meant the participant was required to work in a mental health service at a lower level to obtain the required background to work in the role:

AOD and mental health needed to integrate so I was part of an integration service with my service, and I think there were about seven mental health service workers at the time, and we had quite a good working relationship. I went off and did the dual diagnosis course, and when I came back the health service had changed the protocol for dual diagnosis clinicians, and you had to come from mental health. I had no mental health background at that time, no mental health qualifications, no piece of paper to say that I had Mental Health. So, I then decided, well, if I must – I want to still work with my clients, I know I had dual diagnosis clients here, I know my skills are good, but if I need to – a piece of paper, then I’ll go and do the Mental Health. I went from a Clinical Nurse Consultant down to a registered nurse to do a Masters in Mental Health. (Participant 17)

The notion of the requirement for mental health qualifications after services integrated as being a barrier was echoed by another participant, who spoke of the need for mental health qualifications as being an impediment to finding staff with AOD experience and qualifications:

Because of the mental corporate governance… people think “I don’t have the mental health qualifications so I can’t go for the job,” which is what I would have thought if I was still doing general nursing and I hadn’t transitioned the way I did within the move, and I think that’s a main barrier for us just in the mindset of what’s in the role description. (Participant 29)

Participants also spoke of what they felt was a perceived power and funding imbalance after service integration, with many who had experienced service integration reporting that the ‘mental health way’ of service provision became the dominant model:

We fall under mental health, they’ve amalgamated it, although they like to say as integrate but it’s only when it suits them, when you fall under mental health, that is always a little bit of argy bargy in regard to that. Everything’s about mental health, you’ve got to conform to mental health, and you’ve got to do everything this way but that doesn’t always work for our patients. (Participant 33)

## Discussion

Although the concept of integrating mental health and AOD services aims to improve the comprehensive care of consumers presenting with dual diagnosis, our research indicates that the perception of AOD nurses is that integration will be disadvantageous to their consumers, devalue their specialty and result in reductions in service due to funding cuts. Our findings also indicate that many AOD nurses have experienced service integration that has resulted in job losses, funding reductions and a perception that the consumers they provide care for are stigmatized even within mental health services. Research reflects these concerns, finding consumers with co‐morbidities often experiencing negative attitudes and perceptions (Adams 2008).

Our research also found that there was a perception that skills would need to be improved in both AOD and mental health services to provide comprehensive care to consumers in an integrated model. As has been noted previously, our participants expressed the notion that consumers who are considered to have a primary alcohol and/or drug use disorder would find it difficult to be accepted for treatment into mental health services (Groenkjaer *et al*. 2017; Searby *et al*. 2017). Further, participants felt that seeking help from a mental health service had a consumer perception of ‘medicalising’ drug and alcohol use, and a fear of being ‘involved’ in the mental health system, especially by being recorded on medical record systems associated with mental health. While research shows benefits to integrated care, there is some way to go in ensuring that integrated systems are appropriate to all consumers; this includes core differences in presentations, such as complex trauma, personality disorders, acquired brain injury presentations and physical ill health participants describe as being common in AOD treatment settings.

Philosophical differences in approaches to care were revealed by the participants and uncertainty about each service’s purpose and care delivery perspective. For example, a belief that mental health services were not recovery focussed compared with AOD services; this notion is supported in a scoping review exploring cross‐sector collaboration between mental health and AOD services, where compulsory care and a greater lived experience workforce were identified as key differences between the two services (Minshall *et al*. 2021). It appears that little had been done to engage the participants and share information between services resulting in a fear of change towards integration. Participants could identify benefits of coordination of care for consumers who had both mental health and substance use treatment needs. However, they found it difficult to view how this would work in practice.

International studies show that successful integration often involves the concept of ‘wrap around care’. For example, Hoffman *et al*. (2004) describe integrated services for women who had experienced trauma, addiction and mental ill health in a rural Massachusetts setting. The approach taken here was one of meeting consumer need by providing linkage and access to 12‐step meetings, trauma informed care, recovery and peer assistance, rather than merely ‘bolting on’ a separate system of care. Similar examples have found unmet need and service gaps being a key driving force for integration (Lesage *et al*. 2008). In contrast, Mfoafo‐M’Carthy *et al*. (2021) describe the integration of AOD mental health and problem gambling services in Ontario as being uneven and unequal, with problem gambling services being ‘sidelined’. This finding echoes the concerns of participants in our study who felt that a similar sidelining was a real possibility for the AOD treatment services they worked in, with treatment for mental ill health becoming the key priority and funding driver for integrated services.

Although several models for integrating services exist (Brousselle *et al*., 2007; Rush *et al*. 2008), our research indicates that integration is not a one size fits all situation and requires tailored approaches. Integration could involve the formation of a new blended service with AOD use and mental health disorders treated as one and all clinicians being required to address both areas of care need, echoing the notion of holistic rather than integrated care described by Lee and Allsop (2020) in the background to this paper (Brouselle *et al*. 2010). This approach would arguably result in a reduction in the traditional ‘siloed’ approach of treatment services, and thus a good starting point for any integration effort. We suggest future research of integration models considers the perspectives of clinicians, consumers and the complexities outlined when aiming to provide integrated care.

### Limitations

To our knowledge, this is the first Australian qualitative study of AOD nurses regarding service implementation; however, there are some limitations that need to be considered. Participants in this study provided opinions and experiences that represent subjective views of specific health services and jurisdictions, and therefore may not represent all health services in particular states or territories. Further, participants who had experienced service integration were drawn from one Australian state (Queensland), and therefore experiences in other areas that had moved to integration may differ. Although the focus of our study was nurses, the experiences of other healthcare practitioners may also differ from those of our participants.

Although our study provides insight from AOD nurses into their experiences and perceptions of service integration, further research is needed to overcome barriers of integration, particularly among healthcare consumers seeking treatment from AOD services. As outlined in our participant accounts, these consumers often do not fit the profile of ‘traditional’ mental health services, which may result in an effective service gap. To define these barriers, the opinions of consumers of existing AOD services should be canvassed to obtain the view of service users.

## Conclusion

For the AOD nurses participating in our study who had experienced service integration, it was often viewed as a hostile takeover; the requirements for them to work in their roles had changed, the model of care they worked under was discounted for a mental health‐based model that was felt to be highly risk averse, and their funding streams were reduced. Integration was seen to have greater benefits for mental health services compared with AOD services. Mental Health services were seen to be more dominant, better resourced and funded. Concerns about the focus of care as well as the complexity of care differing between the two services demonstrated a contrast in both philosophical approaches to work with consumers and legislative difference in voluntary versus compulsory care provision.

## Relevance for Clinical Practice

Integration of mental health and AOD services has been a topical issue for several years, and in Victoria recommendations from the Mental Health Royal Commission will drive the move towards integrated services. Through the lens of the AOD nurse, integration has several barriers to providing comprehensive care to consumers who may not necessarily fit the typical profile of those seeking support for mental ill health. There are several barriers that need to be explored and overcome to ensure that integrated services provide care to all consumers currently using mental health and AOD services, respectively.

## ACKNOWLEDGEMENT

The parent study was funded by the Drug and Alcohol Nurses of Australasia (DANA). Open access funding provided by Deakin University.
